# Gelsolin-Independent Podosome Formation in Dendritic Cells

**DOI:** 10.1371/journal.pone.0021615

**Published:** 2011-07-11

**Authors:** Oscar Hammarfjord, Hervé Falet, Christine Gurniak, John H. Hartwig, Robert P. A. Wallin

**Affiliations:** 1 Department of Medicine, Huddinge, Center for Infectious Medicine, Karolinska Institutet, Karolinska University Hospital, Stockholm, Sweden; 2 Translational Medicine Division, Department of Medicine, Brigham and Women's Hospital, Harvard Medical School, Boston, Massachusetts, United States of America; 3 Institute of Genetics, University of Bonn, Bonn, Germany; University of Birmingham, United Kingdom

## Abstract

Podosomes, important structures for adhesion and extracellular matrix degradation, are claimed to be involved in cell migration. In addition, podosomes are also reported to be of importance in tissue remodelling, e.g., in osteoclast-mediated bone resorption. Podosomes are highly dynamic actin-filament scaffolds onto which proteins important for their function, such as matrix metallo-proteases and integrins, attach. The dynamics of the podosomes require the action of many proteins regulating actin assembly and disassembly. One such protein, gelsolin, which associates to podosomes, has been reported to be important for podosome formation and function in osteoclasts. However, podosome-like structures have been reported in gelsolin-deficient dendritic cells, but the identity of these structures was not confirmed, and their dynamics and function was not investigated. Like many other cells, dendritic cells of the immune system also form matrix degrading podosomes. In the present study, we show that dendritic cells form podosomes independently of gelsolin, that there are no major alterations in their dynamics of formation and disassembly, and that they exhibit matrix-degrading function. Furthermore, we found that gelsolin is not required for TLR4-induced podosome disassembly. Thus, the actin cytoskeleton of podosomes involved in dendritic cell extracellular matrix degradation appears to be regulated differently than the cytoskeleton in podosomes of osteoclasts mediating bone resorption.

## Introduction

As sentinels of the immune system, dendritic cells (DC) serve as an important link between innate and adaptive immunity. Immature DC reside in peripheral tissues where they detect invading pathogens using receptors, binding to pathogen molecules, such as the toll-like receptor (TLR) family. Tissue DC undergo a process of maturation that is induced, either spontaneously, or following activation by pathogen recognition. Maturation changes the phenotype of the DC, enabling them to migrate to draining lymph nodes [1]. Activated DC also up-regulate co-stimulatory molecules necessary for T cell activation [2]. By presenting acquired antigens to T cells in the lymph-node they may either induce an adaptive immune response under inflammatory conditions, or induce tolerance under steady state conditions [2]. Thus, activation and migration from peripheral tissues to the lymph node is an essential step in the life cycle of DC.

To enable migration, DC employ matrix metalloproteinases (MMPs), e.g., MMP14, localized at podosomes, to degrade the connective tissue matrix [3]–[5]. It has been hypothesized that DC use podosomes as pathfinders, enabling degradation of extracellular matrix at the front of the moving cell, facilitating migration into and through dense connective tissue [5]. The podosomes in DC are also regulated by TLR signalling. Activation of DC with TLR ligands from microbes lead to rapid and transient disassembly of podosomes and concomitant loss of migratory capacity during the first hours of activation [4], [6]. This may serve to transiently stop the DC from migrating from the area of pathogen encounter and to focus instead on acquiring antigens via endocytosis and/or phagocytosis. However, much still remains to be learned about how podosome formation and function are regulated in DC and other cells [7]. Podosomes, found in many myeloid cell types, and also in some epithelial cells, are F-actin enriched adhesion structures that degrade extracellular matrix during cell migration, trans-cellular migration and bone resorption [8]–[11]. The internal podosome scaffold is a radial array of F-actin, actin-associated proteins, and signalling transducers [12]. The podosomal scaffolds are highly dynamic and many proteins, important for actin assembly/disassembly, such as the arp2/3 complex, Cdc42, WASP, cofilin, and cortactin, have been reported to be important for podosome formation [8], [10], [13]–[17]. The actin-severing and capping protein gelsolin is another actin-cytoskeleton regulating protein that has been reported to localize to podosomes in several cell types, and to be important for formation and function of podosomes in osteclasts [8], [18].

West and co-workers reported podosome-like structures in gelsolin-deficient DC, however, no further characterization were made of these structures [19]. Since gelsolin have been claimed to be essential for podosome formation and function in osteoclasts, and has been cited as important for podosomes in several papers [12], [20]–[24], we decided to study these podosome-like structures in DC, to clarify if these are indeed functional podosomes.

## Materials and Methods

### Ethics Statement

Harvesting of murine bone marrow from sacrificed mice was approved by the Stockholm animal ethical committee north (Stockholms Norra Djurförsöksetiska Nämnd). Approval ID number: N269/07.

### Mice and bone marrow-derived DC

The mice used were six to ten weeks old C57BL/6 and gelsolin knock-out on C57BL/6 background females housed under specific pathogen-free conditions at the animal facility at the Translational Medicine Division, Department of Medicine, Brigham and Women's Hospital, Harvard Medical School or at the Institute of Genetics, University of Bonn. The mice were sacrificed by cervical dislocation. The gelsolin knock-out mice strain used in the present study is the same strain used in previous studies by Chellaiah *et al.* [8] and West *et al.* [19]. All mice were genotyped before use. Murine bone marrow-derived DC (BMDC) was generated from femurs and tibia as previously described[25]. Briefly, cells from the marrow of C57BL/6 mice were grown in DC culture medium (DMEM containing 4.5 g/l glucose, l-glutamine, pyruvate, streptomycin (Invitrogen), 10% fetal bovine serum (HyClone), and 10 ng/ml granulocyte macrophage-colony stimulating factor (GM-CSF) (Peprotech). On day 3, 1 ml fresh medium with GM-CSF was added to a final volume of 3 ml. On day 6, the cells were harvested for use.

### Western blot analysis

To confirm presence of gelsolin the wild-type DC as well as absence of the gelsolin in the gelsolin-deficient DC, western blot analysis was performed. Day 6 DC were spun down and immediately resuspended in pre-heated sodium dodecyl sulfate (SDS)–sample buffer (Invitrogen) containing 50 µM Dithiothreitol (DTT). The samples were heated to 90°C and then frozen and thawed twice to degrade DNA. The proteins in the samples were separated on a 10–20% NOVEX SDS-polyacrylamide gel (Invitrogen) and transferred to PVDF membranes (Invitrogen). The membranes were blocked with 5% bovine serum albumin (Invitrogen) in phosphate buffered saline (PBS) and incubated with goat anti-gelsolin (n-18) (Santa Cruz Biotechnology) at 4°C overnight. After washing, the blot was incubated with horseradish peroxidase (HRP)-coupled goat anti-rabbit antisera (Jackson ImmunoResearch Laboratories) and was developed with Super Signal West Pico Chemiluminescent Substrate (Pierce). To confirm equal loading, the membrane was stripped using Re-Blot Plus Strong (Millipore), blocked as described above and incubated with mouse anti-actin (C4) (Millipore) for 1 h in room temperature. After washing, the blot was incubated for 1 h in room temperature with horseradish peroxidase (HRP)-coupled rabbit anti-mouse antisera (Jackson ImmunoResearch Laboratories) and was developed as described above. The chemiluminescent signal was captured with a digital LAS 4000 system (Fuji Film Life Science).

### Microscopy

WT and gelsolin-deficient DC were plated on glass coverslips in 24 well plates. The cells were then incubated for 2 hours before cells were incubated with or without 100 ng/ml LPS (Sigma-Aldrich) for 20 min. The cells were fixed for 15 min with 4% paraformaldehyde (PFA) that was pre-warmed to 37°C. After a step of washing, the cells were permeabilized with 0.1% Tx100 for 10 minutes and vinculin was stained over night (in PBS 0.01 Tx100, 1%BSA) using a FITC anti-vinculin antibody (Sigma-Aldrich) followed by a 30 min staining of the actin cytoskeleton was stained with TRITC-phalloidin (Sigma-Aldrich) the stainings were followed by extensive wash using TBS with 1% BSA and 0.01%Tx100. The coverslips were mounted with ProLong Gold antifade (Invitrogen) containing DAPI. The actin cytoskeleton morphology and podosome structures were observed with a Delta Vision Spectris microscope (Applied Precision) and analyzed using the cell counter plugin for ImageJ (http://imagej.nih.gov/ij/). The percentage of DC that exhibited podosome structures was calculated. More the 30 fields containing an average of 6 cells per field were analyzed in each group. Podosome ultra-structure was assessed using TEM. Replicas of the cytoskeleton of DC were made as described by Hartwig [26], with some modifications. Briefly, ventral membrane preparations were made by mechanical fragmentation of the cells (“unroofing”) followed by glutaraldehyde fixation in PHEM buffer. The samples were rapidly frozen on helium-cooled block, freeze-dried at −90C, rotary shadowed with either platinum to tantalum-tungsten (∼1–5 nm) and stabilized with 5 nm of carbon without rotation. The replicas were picked up on grids and viewed in the EM at 80 kV.

### Extra-cellular matrix (ECM)-degrading capacity

Oregon green gelatin (Molecular Probes, Eugene, OR, USA) was added to sterile coverslips in 24-well plates and incubated for 30 min. The gelatin-coated coverslips were then stabilized with 4% PFA for 15 min and washed extensively with PBS. After a last wash with DC medium, the coverslips were incubated in complete medium for 30 min to block remaining reactive PFA. After another wash, 500 µl of DC cell suspension at a concentration of 0.5×10^6^ cells/ml was added. The cells were then incubated overnight before they were fixed with 4% PFA and stained with phalloidin (Alexa Fluor 555, Invitrogen). The coverslips were mounted with ProLong Gold antifade containing DAPI. Images were acquired with a Delta Vision Spectris microscope (Applied Precision), and extracellular matrix-degrading capacity was analysed with ImageJ (http://imagej.nih.gov/ij/). The Oregon green gelatin images were analyzed automatically using a ImageJ macro that performs Otsu thresholding of the image and measures the percent of the area where the oregon green gelatin signal is below the threshold (supplementary methods S1). Another macro was written to automatically count the number of nuclei in the image files. The average of more then 390 fields (with a total of at least 2000 cells) was calculated for DC from both gelsolin-deficient and wild-type mice.

### Podosome dynamics

To visualize podosomes as they assemble and disassemble, DC from gelsolin-deficient and wild-type mice were transduced with retrovirus coding for GFP-actin. Cells were seeded in glass-bottom dishes (Mattek) and observed every 10 sec for a period of 15 min with the DeltaVision Spectris live cell imaging system (Applied Precision). 14 cells of each type were imaged and representative image montages and videos are presented. Lifetime of more than 100 individual podosomes was measured in these videos using MTrackJ analysis plugin for ImageJ (http://imagej.nih.gov/ij/).

### Migration assay

Transwell filters were coated with a thin layer, covering the filter area, of matrigel (BD bioscience) and left to stabilize for 30 min at 37°C. DC were harvested on day 6 and seeded on matrigel coated or uncoated transwell filters, (6-well format, 3-µm pore size; BD Labware, Bedford. MA, USA) at a density of 2–3×10^6^ cells/well, and incubated the time indicated in the figure. Cells that had migrated to the bottom compartment were counted in Bürker-chambers using trypan blue exclusion and analyzed by using flow-cytometry. Cells were stained with anti-CD11c Alexa 647 (e-bioscience). The staining was performed on ice followed by extensive wash with 1% FBS and 0.1% NaN_3_ in PBS. Cells were analyzed on a FACSCalibur cytometer using CellQuest software.

### Statistical analysis

Statistical analysis was performed using the two-tailed nonparametric Mann-Whitney test in Prism (GraphPad 5.0a) software. Results were considered significant at P<0.05. *** denotes P<0.001. Standard error bars are used.

## Results

### Podosome formation in DC is independent of gelsolin

**Figure 1:**
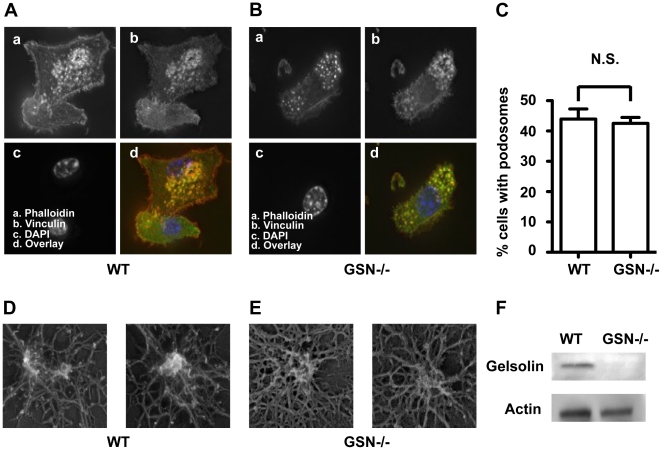
Podosome formation in gelsolin deficient DC. Immuno-fluorescence microscopy of (A) wild-type (WT) DC and (B) gelsolin deficient (GSN−/−) DC. a-d denotes staining with: phalloidin, vinculin, dapi and overlay respectively. (C) Quantification of the number of wild-type (WT) and gelsolin deficient (GSN−/−) DC exhibiting podosome clusters. Average of more than 30 fields (>150 cells) per cell type. N.S. denotes non significant. Podosome ultra-structure, of (D) wild-type (WT) and (E) gelsolin deficient (GSN−/−) DC, visualised by electron microscopy. (F) Western blot analysis of gelsolin protein expression in wild-type (WT) and gelsolin deficient (GSN−/−) DC.

To confirm that the phalloidin stained structures observed by West *et al.* [19] in gelsolin-deficient DC are indeed podosomes, we stained DC from gelsolin-deficient mice with phalloidin and an antibody specific for the podosome-associated protein vinculin [23]. Staining of these DC resulted in a typical vinculin staining pattern, localising around the dense phalloidin stained actin core on the ventral cell surface of DC derived from both gelsolin-deficient and wild-type mice (Fig. 1A and B). The proportion of cells exhibiting podosome structures was quantified and no difference between gelsolin-deficient and wild-type DC was found (Fig. 1C). We also used electron microscopy to visualise the podosomes in DC from gelsolin-deficient and wild-type mice. By “unroofing” the DC, fixing and metal-shadowing the structures associated with the ventral membrane, electron microscopy visualisation of podosomes in the cell-membrane associated actin cytoskeleton was possible. The podosomes were clearly identifiable in both the wild-type and the gelsolin-deficient DC (Fig. 1D and E). In the examples shown, the density and branching of actin filaments in the podosomes may differ somewhat between the wild-type and the gelsolin-deficient DC. However, considering that these are highly dynamic structures, we cannot from the current data conclude that their structure differ greatly. The bone marrow DC generated from the gelsolin mutant mice was confirmed to be gelsolin negative by western blotting analysis (Fig. 1F). We thus conclude that podosome formation is independent of gelsolin in mouse bone marrow derived DC.

### Podosome dynamics in DC is not greatly altered in the absence of gelsolin

**Figure 2:**
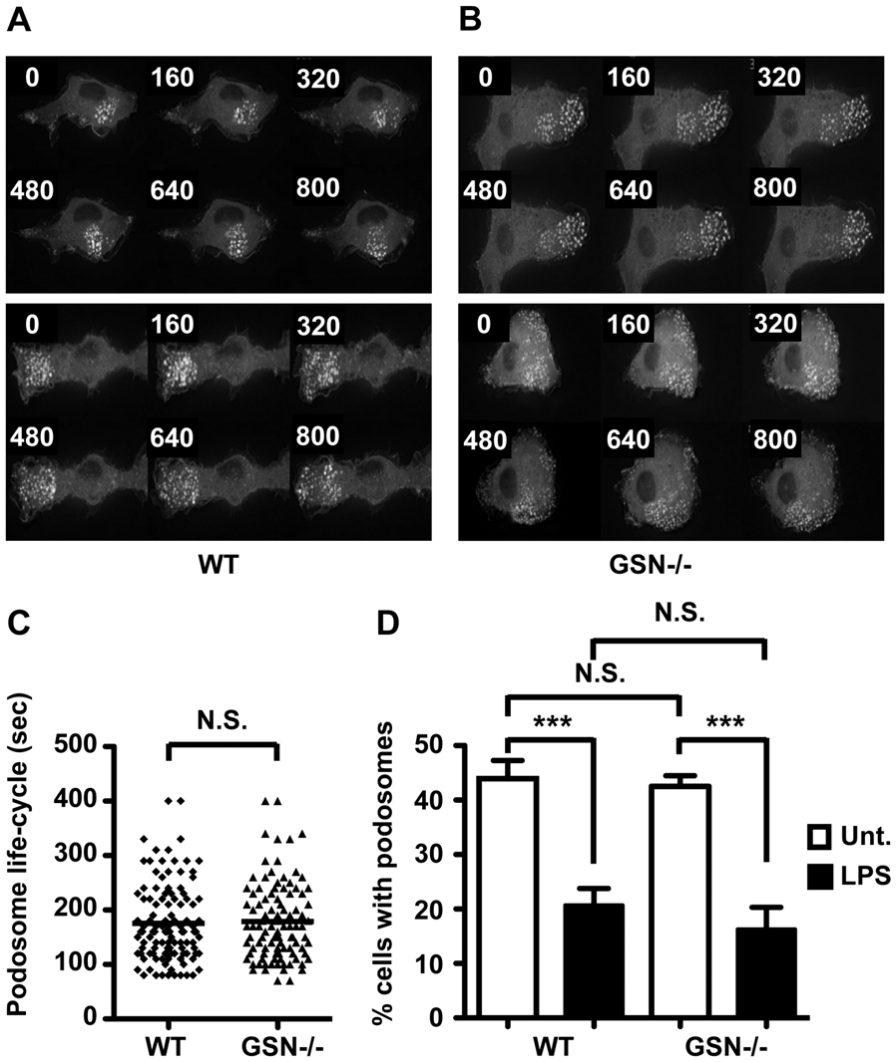
Podosome dynamics in gelsolin deficient DC. Image panels of two representative time-lapse microscopy sequences each of GFP-actin expressing, (A) wild-type (WT) DC and (B) gelsolin deficient (GSN−/−) DC (numbers indicate time in seconds). (C) The podosome life-cycle in seconds was quantified in time-lapse microscopy sequences. (D) Quantification of the number of, wild-type (WT) and gelsolin deficient (GSN−/−), DC exhibiting podosome clusters in untreated (Unt.) and LPS treated DC. The data presented of the untreated DC is the same as in Fig. 1. Average of more than 30 fields (>150 cells) per cell type. Error bars indicate standard error. ***P<0.001, N.S. denotes non-significant.

Having established that formation of podosomes in DC is independent of gelsolin, we next studied the dynamics of podosome assembly and disassembly in gelsolin-deficient DC. To visualize the dynamic process of podosome assembly and disassembly, gelsolin-deficient and wild-type DC were transduced with a retro-viral vector coding for GFP-actin, after which the cells were studied using live cell imaging. This revealed that the dynamics of podosome assembly and disassembly in DC is seemingly unaffected by the lack of gelsolin (Fig. 2A and B, Supplementary movie S1 and S2). Moreover, quantitative analysis of the podosome life-time showed no differences between gelsolin deficient DC and control DC (Fig. 2C). We have previously shown that TLR stimulation of DC result in a transient disassembly of podosomes [6]. To investigate if the TLR-stimulated disassembly of podosomes requires the activity of gelsolin, we quantified the proportion of gelsolin- deficient DC exhibiting podosomes after 20 minutes of TLR4 stimulation and compared this to wild-type DC (Fig. 2D). These data showed that podosome disassembly induced by TLR signalling does not require the actin severing activity of gelsolin. Thus, podosome dynamics, in DC, seem not to be greatly altered in the absence of gelsolin.

### Podosome function in DC is independent of gelsolin

**Figure 3:**
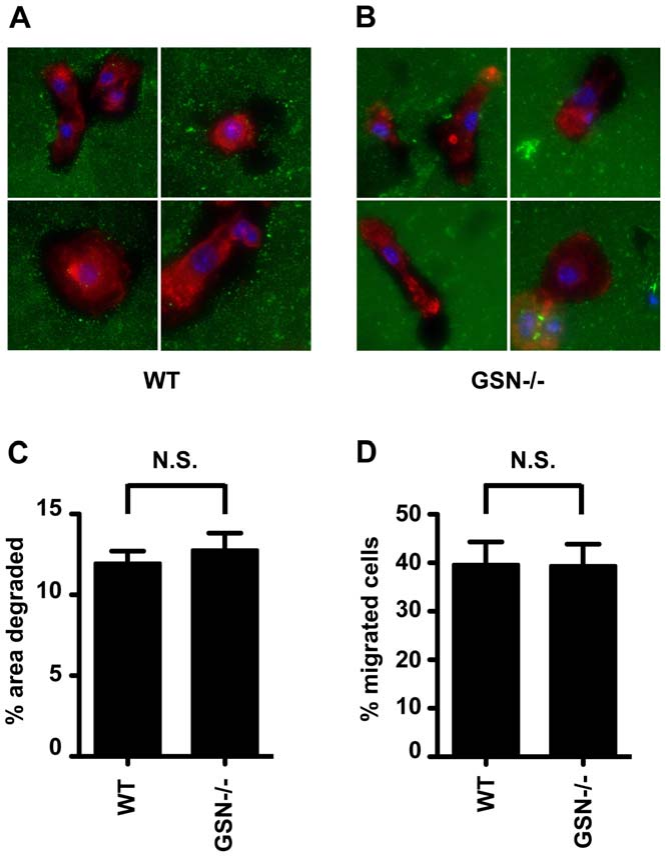
Podosome function in gelsolin deficient dendritic cells. Four representative images of (A) wild-type (WT) DC or (B) gelsolin deficient (GSN −/−) DC degrading oregon-green labeled gelatin, coated on glass cover slips. Phalloidin staining is shown in red, DAPI staining of the DNA in blue, and Oregon-green labeled gelatin in green. (C) Quantification of the area of Oregon-green labeled gelatin degraded by wild-type (WT) and gelsolin deficient (GSN −/−) DC (>350 fields containing in total around 2000 cells were analyzed per cell type) using Image J software. (D) Migration of wild-type (WT) and gelsolin deficient (GSN −/−) DC through matrigel-coated transwells. The percentage of DC that migrated to the bottom well overnight (12 h) is shown. Migration experiments were performed in triplicate (two experiments). Error bars indicate standard error. N.S., indicate non-significant.

To address whether the podosomes observed in the gelsolin-deficient DC are impaired in function, as been shown in osteoclasts, the capacity to degrade extracellular matrix was evaluated. DC were seeded on glass coverslips coated with fluorescent Oregon-green gelatin and incubated over night before the samples were fixed and stained with phalloidin. Fluorescence microscopy analysis showed that gelsolin-deficient DC were able to degrade the gelatin (Fig. 3A and B). Quantitative analysis of the total area of degraded gelatin, performed on more than 390 image fields for each cell-type concluded that there were no major differences in the capacity of gelsolin-deficient DC and wild-type DC in the degradation of extracellular matrix (Fig. 3C). To study if gelsolin-deficient DC can utilize the capacity to degrade extracellular matrix in order to migrate through dense tissue-matrix, a transwell migration assay coated with a dense layer of matrigel was used. We have previously shown that DC migration in this model correlates well with the matrix degrading activity of their podosomes [27]. We found that gelsolin-deficient DC and wild-type DC migrate equally well from the matrigel-coated top-insert to the bottom compartment (Fig. 3D). Thus, podosomes in gelsolin deficient DC are functional in matrix degradation.

## Discussion

Podosomes exist in a wide array of different cell types, and are involved in distinct processes such degradation of extracellular matrix, bone degradation mediated by osteclasts [8], [28], and lymphocyte trans-cellular migration [9]. Current podosome research often extrapolates conclusions made from studies in one cell type, to podosomes in general, and so far differences in the regulation of these structures has not been reported. Gelsolin is a protein frequently mentioned in the literature as one of the key proteins involved in podosome dynamics and function [12], [20]–[24]. Gelsolin was found to localize to podosomes already in the early analysis of these structures, and gelsolin was also found in dendritic cell podosomes [5], [18]. Chellaiah and co-workers then showed that gelsolin-deficient osteoclasts form abnormal podosomes that are compromised in function, which leads to impaired bone resorption [8], [18]. Hu and co-workers have also published a theoretical model for the regulation of podosome dynamics that is dependent on the severing of actin filaments by gelsolin [24]. However, in a study of membrane ruffling, macropinocytosis, and antigen processing in gelsolin deficient DC (processes that all turned out to be independent of gelsolin), West and co-workers showed that phalloidin stained gelsolin deficient DC exhibit structures similar to podosomes, however, these structures were not further investigated [19]. The results in the present manuscript confirm that the actin-rich structures observed by West *et al.* [19], are indeed podosomes. We also extend the analysis and show that the dynamics and function of these podosomes in DC is not greatly altered in the absence of the gelsolin protein.

What then may be the explanation for the differential dependence of gelsolin in DC and osteoclast podosomes? One speculation may be that they are rather different structures built from similar components, i.e., the individual podosomes, and that it may be the supra-molecular organisation of the podosomes into podosome rings, constituting the sealing zone, that requires gelsolin. Dendritic cells and also macrophages tend to organise their podosomes into clusters of podosomes at what looks like random locations on the attachment surface of the cell. These clusters are dynamic and may change location as the cell changes shape while the sealing zones of osteoclasts are likely more static. Another speculation is that another member of the gelsolin protein family is differentially expressed in DC and osteoclasts and compensates for the lack of gelsolin. The mammalian gelsolin superfamily of proteins consists, in addition to gelsolin, of: CapG, adseverin, villin, advillin, supervillin/archvillin, and flightless-1 homologue (FLII) [29], [30]. The expression of Villin seems restricted to epithelial cells of certain organs like the intestine and kidney [30]. Also the expression of advillin seems absent from spleen, bone marrow and leukocytes [31]. Adseverin, which has the highest similarity to gelsolin, may be expressed in macrophage type cells according to The Human Protein Atlas project (proteinatlas.org), however this remains to be confirmed in a publication. Supervillin/Archvillin (probably Supervillin since Archvillin seems to have a muscle specific expression) was recently described by proteomics analysis to be expressed in macrophages [32], [33]. FLII is expressed in many types of tissues including leukocytes (proteinatlas.org) and is the only gelsolin family member essential for development [34]. CapG is important for macrophage actin dynamics such as ruffling [35]. CapG is also expressed in osteoclasts, (United States Patent Application 20030017502), so differences in CapG expression is thus unlikely to lead to the difference in requirement for gelsolin function. Also, CapG lacks actin-severing function and mediates its function by capping actin fillaments [35]. No differences in the expression of these proteins are currently known between DC and osteoclasts that could indicate a compensation effect for the lack of gelsolin. In fact little or nothing is known about the expression of these proteins in DC and osteoclasts. It could thus be interesting in future studies to investigate the function of adseverin, supervillin, FLII, and CapG in the regulation of podosome function.

In a previous study we discovered that podosomes were transiently disassembled after engagement of TLR4 with LPS [6]. One could thus speculate that activation of the actin filament severing capacity of gelsolin could play a role in the rapid disassembly of the podosomes seen after TLR activation. Our data presented here, however, show that the podosomes are efficiently disassembled in the gelsolin-deficient DC, disproving this hypothesis.

In summary, the actin severing protein gelsolin is dispensable for podosome formation, dynamics, and function in DC. Our data indicate that podosomes in different cell types may be differently regulated.

## Supporting Information

Methods S1 ImageJ macros enabling automatic image analysis of the area of oregon green gelatin break-down and counting number of nuclei from a library of Delta Vision microscopy images.
(DOC)

Movie S1 Podosome dynamics in gelsolin deficient DC. Montage of 4 time-lapse microscopy sequences of GFP-actin expressing gelsolin deficient DC (numbers indicate time in seconds).
(AVI)

Movie S2 Podosome dynamics in wild type DC. Montage of 4 time-lapse microscopy sequences of GFP-actin expressing wild type DC (numbers indicate time in seconds).
(AVI)

## References

[1] Banchereau J, Steinman RM (1998). Dendritic cells and the control of immunity.. Nature.

[2] Lanzavecchia A, Sallusto F (2001). Regulation of T cell immunity by dendritic cells.. Cell.

[3] Calle Y, Carragher NO, Thrasher AJ, Jones GE (2006). Inhibition of calpain stabilises podosomes and impairs dendritic cell motility.. J Cell Sci.

[4] West MA, Prescott AR, Chan KM, Zhou Z, Rose-John S (2008). TLR ligand-induced podosome disassembly in dendritic cells is ADAM17 dependent.. J Cell Biol.

[5] Gawden-Bone C, Zhou Z, King E, Prescott A, Watts C (2010). Dendritic cell podosomes are protrusive and invade the extracellular matrix using metalloproteinase MMP-14.. J Cell Sci.

[6] West MA, Wallin RP, Matthews SP, Svensson HG, Zaru R (2004). Enhanced dendritic cell antigen capture via toll-like receptor-induced actin remodeling.. Science.

[7] Burns S, Thrasher AJ, Blundell MP, Machesky L, Jones GE (2001). Configuration of human dendritic cell cytoskeleton by Rho GTPases, the WAS protein, and differentiation.. Blood.

[8] Chellaiah M, Kizer N, Silva M, Alvarez U, Kwiatkowski D (2000). Gelsolin deficiency blocks podosome assembly and produces increased bone mass and strength.. J Cell Biol.

[9] Carman CV, Sage PT, Sciuto TE, de la Fuente MA, Geha RS (2007). Transcellular diapedesis is initiated by invasive podosomes.. Immunity.

[10] Dovas A, Gevrey JC, Grossi A, Park H, Abou-Kheir W (2009). Regulation of podosome dynamics by WASp phosphorylation: implication in matrix degradation and chemotaxis in macrophages.. J Cell Sci.

[11] Albiges-Rizo C, Destaing O, Fourcade B, Planus E, Block MR (2009). Actin machinery and mechanosensitivity in invadopodia, podosomes and focal adhesions.. J Cell Sci.

[12] Saltel F, Daubon T, Juin A, Ganuza IE, Veillat V, et al..

[13] Kaverina I, Stradal TE, Gimona M (2003). Podosome formation in cultured A7r5 vascular smooth muscle cells requires Arp2/3-dependent de-novo actin polymerization at discrete microdomains.. J Cell Sci.

[14] Moreau V, Tatin F, Varon C, Genot E (2003). Actin can reorganize into podosomes in aortic endothelial cells, a process controlled by Cdc42 and RhoA.. Mol Cell Biol.

[15] Linder S, Nelson D, Weiss M, Aepfelbacher M (1999). Wiskott-Aldrich syndrome protein regulates podosomes in primary human macrophages.. Proc Natl Acad Sci U S A.

[16] Webb BA, Eves R, Mak AS (2006). Cortactin regulates podosome formation: roles of the protein interaction domains.. Exp Cell Res.

[17] Bowden ET, Barth M, Thomas D, Glazer RI, Mueller SC (1999). An invasion-related complex of cortactin, paxillin and PKCmu associates with invadopodia at sites of extracellular matrix degradation.. Oncogene.

[18] Gavazzi I, Nermut MV, Marchisio PC (1989). Ultrastructure and gold-immunolabelling of cell-substratum adhesions (podosomes) in RSV-transformed BHK cells.. J Cell Sci 94 (Pt.

[19] West MA, Antoniou AN, Prescott AR, Azuma T, Kwiatkowski DJ (1999). Membrane ruffling, macropinocytosis and antigen presentation in the absence of gelsolin in murine dendritic cells.. Eur J Immunol.

[20] Ma T, Sadashivaiah K, Chellaiah MA Regulation of sealing ring formation by L-plastin and cortactin in osteoclasts..

[21] Ma T, Samanna V, Chellaiah MA (2008). Dramatic inhibition of osteoclast sealing ring formation and bone resorption in vitro by a WASP-peptide containing pTyr294 amino acid.. J Mol Signal.

[22] Crowley JL, Smith TC, Fang Z, Takizawa N, Luna EJ (2009). Supervillin reorganizes the actin cytoskeleton and increases invadopodial efficiency.. Mol Biol Cell.

[23] Van Goethem E, Guiet R, Balor S, Charriere GM, Poincloux R Macrophage podosomes go 3D.. Eur J Cell Biol.

[24] Hu S, Biben T, Wang X, Jurdic P, Geminard JC Internal dynamics of actin structures involved in the cell motility and adhesion: Modeling of the podosomes at the molecular level..

[25] Inaba K, Inaba M, Romani N, Aya H, Deguchi M (1992). Generation of large numbers of dendritic cells from mouse bone marrow cultures supplemented with granulocyte/macrophage colony-stimulating factor.. J Exp Med.

[26] Hartwig JH (1992). Mechanisms of actin rearrangements mediating platelet activation.. J Cell Biol.

[27] Hammarfjord O, Wallin RP (2010). Dendritic cell function at low physiological temperature.. J Leukoc Biol.

[28] Gimona M, Buccione R, Courtneidge SA, Linder S (2008). Assembly and biological role of podosomes and invadopodia.. Curr Opin Cell Biol.

[29] Silacci P, Mazzolai L, Gauci C, Stergiopulos N, Yin HL (2004). Gelsolin superfamily proteins: key regulators of cellular functions.. Cell Mol Life Sci.

[30] Khurana S, George SP (2008). Regulation of cell structure and function by actin-binding proteins: villin's perspective.. FEBS Lett.

[31] Marks PW, Arai M, Bandura JL, Kwiatkowski DJ (1998). Advillin (p92): a new member of the gelsolin/villin family of actin regulatory proteins.. J Cell Sci.

[32] Trost M, English L, Lemieux S, Courcelles M, Desjardins M (2009). The phagosomal proteome in interferon-gamma-activated macrophages.. Immunity.

[33] Weintz G, Olsen JV, Fruhauf K, Niedzielska M, Amit I (2010). The phosphoproteome of toll-like receptor-activated macrophages.. Mol Syst Biol.

[34] Campbell HD, Fountain S, McLennan IS, Berven LA, Crouch MF (2002). Fliih, a gelsolin-related cytoskeletal regulator essential for early mammalian embryonic development.. Mol Cell Biol.

[35] Witke W, Li W, Kwiatkowski DJ, Southwick FS (2001). Comparisons of CapG and gelsolin-null macrophages: demonstration of a unique role for CapG in receptor-mediated ruffling, phagocytosis, and vesicle rocketing.. J Cell Biol.

