# Supplementary material for: Gelsolin-Independent Podosome Formation in Dendritic Cells
Source: PLoS One. 2011 Jul 11;6(7):e21615. doi: 10.1371/journal.pone.0021615 (PMC3136926; doi:10.1371/journal.pone.0021615)
Supplement: Methods S1 — ImageJ macros enabling automatic image analysis of the area of oregon green gelatin break-down and counting number of nuclei from a library of Delta Vision microscopy images. (DOC) [file pone.0021615.s001.doc]

**Supplementary material**

ImageJ macro quantifying oregon green gelatin breakdown:

dir1 = getDirectory("Choose Source Directory ");

dir2 = getDirectory("Choose Destination Directory ");

list = getFileList(dir1);

setBatchMode(true);

for (i=0; i<list.length; i++) {

showProgress(i+1, list.length);

A = dir1+list[i];

print(A);

run("Deltavision Opener", "open=A");

B = getInfo("image.filename");

C = substring (B, 0, lastIndexOf(B,"."));

print(C);

run("Stack to Images");

selectWindow(C+"-0001");

setAutoThreshold("Otsu dark");

run("Measure");

selectWindow(C+"-0001");

saveAs("Tiff", A);

run("Close All");

}

ImageJ macro counting number of nuclei:

dir1 = getDirectory("Choose Source Directory ");

dir2 = getDirectory("Choose Destination Directory ");

list = getFileList(dir1);

setBatchMode(true);

for (i=0; i<list.length; i++) {

showProgress(i+1, list.length);

A = dir1+list[i];

run("Deltavision Opener", "open=A");

B = getInfo("image.filename");

C = substring (B, 0, lastIndexOf(B,"."));

print(C);

run("Stack to Images");

selectWindow(C+"-0003");

run("8-bit");

run("Nucleus Counter", "smallest=1000 largest=10000 threshold=Otsu smooth=[Mean 5x5] subtract show");

selectWindow(C+"-0003");

saveAs("Tiff", A);

run("Close All");

}
